# The effectiveness of Sumbawa fermented mare’s milk powder with gum arabic [*Acacia senegal* (L.) Willd] microencapsulant as a modified traditional healthy food

**DOI:** 10.5455/javar.2025.l925

**Published:** 2025-06-02

**Authors:** Arif Hendra Utama, Khothibul Umam Al Awwaly, Lilik Eka Radiati

**Affiliations:** Faculty of Animal Science, Brawijaya University, Malang, Indonesia

**Keywords:** Sumbawa fermented mare’s milk, gum Arabic (*Acacia senegal* (L.) Willd, microencapsulant, milk powder

## Abstract

**Objective:**

This study is to investigate the concept of gum arabic (*Acacia senegal* (L.) Willd.) as microencapsulation for traditional healthy food modified with the concept of powder drying with that derived from fermented Sumbawa mare’s milk with the concept of improving the integrity of healthy processed food products.

**Materials and Methods:**

The use of gum arabic (*Acacia senegal* (L.) Willd.) as a microencapsulant of Sumbawa mare’s milk powder with treatments (T1: 20%, T2: 24%, T3: 28%, and T4: 32%). Fermented Sumbawa mare’s milk samples were dried using the oven vacuum foam drying method with gum arabic binder at 70°C for 420 min. Evaluation of product test results by analyzing antibacterial, antioxidant, *Escherichia coli* concentration, protein content, and microstructure.

**Results:**

Based on the treatment of the results of the research, it has been significant (*p* < 0.01) that Sumbawa mare’s milk powder with the use of gum arabic as a microencapsulant known antibacterial test (*Lactobacillus casei* ATCC 393:4.56 mm, *Bacillus subtilis* ATCC 6633:7.36 mm, *E. coli* ATCC 25922:12.25 mm, and *Pseudomonas aeruginosa* ATCC 15442:12.2 mm), as well as an antioxidant test, which has a value of (46.13 ± 0.73c), microstructure, and protein content using liquid chromatography-mass spectrometry/mass spectrometry analysis, which is reinforced with health pharmaceutical information with literature studies. The *E. coli* test (food quality test) had a value of 0 CFU/gm, with the results shown in treatment T4 (32%).

**Conclusion:**

The use of gum Arabic as a natural additive microencapsulant can be promising in the production of Sumbawa mare’s milk powder. The results obtained have a significant effect on increasing protection and reducing the risk of unwanted pathogenic microbacterial contamination, can reduce antioxidant levels, and provide knowledge for the general public in improving the quality and healthy nutrition of traditional foods.

## Introduction

Mare’s milk has a high content of probiotics and peptides, especially horse milk that has undergone a natural fermentation process or has been fermented by humans, such as koumiss and airag, so it has a higher probiotic and bioactive content that can be used for the treatment of various disorders, such as digestion, antimicrobial, and immunity [1]. One of the products in Indonesia that has not been widely exposed is Sumbawa mare’s milk. It has long been consumed by the people of Sumbawa, which has content and features as a health product. Sumbawa people believe that the sourer the flavor, the higher the level of nutrition, and it is included in products that are accidentally fermented [2]. The most important content in fermented Sumbawa mare’s milk is the natural microorganisms that are naturally affiliated with it, thus affecting its nutrition.

One of the species affiliated with Sumbawa mare’s milk naturally is *Lactobacillus* sp., which plays a role in the development in terms of texture, aroma, and acidity and is believed to be the main source that affects and benefits human health. Fermented Sumbawa mare’s milk has the advantage of containing naturally beneficial active micro-bacteria in its product, so it is called a natural source of probiotics. As a source of natural probiotics, Sumbawa mare’s milk has advantages for antibacterial sources such as *Salmonella* sp. and *Staphylococcus aureus* [3]. In addition, the results of mare’s milk can reduce the number of pathogenic microorganisms, so it can be signaled to be one of the health products [4,5]. The most important ingredients in fermented products from mare’s milk are such as active peptides; the bioactive content of milk protein in particular plays an important role in food processing applications due to its water binding, viscosity, gelation, emulsification, and foaming capabilities. In addition, mare’s milk fermented products are reported to that the following gross composition on average: 2.14% protein, 1.21% fat, and 6.37% lactose and other bioactive molecules [6].

The use of developing technology such as microparticles in a food product is one of the developing projects echoed or modified around the world in various fields such as food, medicine, processed beverages, and environmental remediation processing. The application of microencapsulation is a strategy to improve the quality and stability of the milk and avoid clumping and separation from added ingredients like gum Arabic. Gum arabic (*Acacia senegal* (L.) Willd) is a polymer used as a filler, stabilizer, thickener, and encapsulant agent in dry food products [7]. Furthermore, the method of producing and combining microcapsulants with food-grade additives could enable the development of materials with better mechanical and functional properties in the field of medicine and in the context of long-lasting food processing [8]. This is due to the formation of a protective barrier around the milk powder particles during the microencapsulation process with gum arabic (*Acacia senegal* (L.) Willd).

Vacuum drying allows evaporation of water at lower boiling point temperatures ranging from 60°C to 80°C, depending on the product being dried. The aim of such drying is to reduce oxygen levels and heat-sensitive food components such as enzymes, proteins, and peptides. LAB bacteria can be vacuum-dried successfully by minimizing their losses [9]. Drying processes are intended and applied for the preservation and extension of the shelf life of food products by reducing the moisture content to improve and enable the minimization of microbiological and chemical degradation reactions and at the same time reduce storage and transportation expenses [10].

Therefore, this study aims to test and determine the effect of the use of natural microencapsulant gum arabic (*Acacia senegal* (L.) Willd) on fermented Sumbawa mare’s milk powder with tests carried out such as antibacterial, antioxidant, and microstructure and reinforced with protein content tests with pharmaceutical information in the literature. The results of this analysis aim to identify the benefits of gum arabic as a microencapsulant in processing milk powder to improve the health benefits and functional foods derived from dairy products, especially traditional fermented preparations such as Sumbawa mare’s milk powder. This could make it a valuable subject for research and development for determining the effects of functional foods and nutraceuticals on human health.

## Materials and Methods

### Ethical approval

The work has been conducted following the ethical standards set by the university. However, no formal approval was needed for conducting this experiment.

### Location and timing

This research was conducted at the Animal Product Technology Laboratory, Faculty of Animal Husbandry, Universitas Brawijaya for the preparation of Sumbawa mare’s milk powder, *Escherichia coli* count, and antibacterial activity. The antioxidant IC_50_ activity test was conducted at the Animal Husbandry Laboratory of Muhammadiyah University of Malang. The analysis of the microstructure of Sumbawa horse milk powder with field emission scanning electron microscopy with energy dispersive X-ray spectroscopy (FESEM)/(EDX) and protein content with liquid chromatography-mass spectrometry/mass spectrometry (LCMS/MS) was carried out at the Integrated Research and Testing Laboratory (LPPT), Universitas Gadjah Mada. We conducted the research from September 2023 to January 2024.

### Materials and equipment

Mare’s milk was obtained from Mr. Tamrin’s partnership with Curi Mori Sumbawa mare’s milk brand in West Nusa Tenggara. Food-specific test bacteria *Lactobacillus casei* ATCC 393, *Bacillus subtilis* ATCC 6633, *E. coli* ATCC 25922, and *Pseudomonas aeruginosa* ATCC 15442 were obtained at the Food Microbiology Laboratory, Faculty of Agricultural Technology, Universitas Brawijaya. The following products were acquired from Oxoid: DeMann broth, Regosa Sharp agar, and Nutrient Agar (Nurra Gemilang Chemical Store under license from Oxoid, Basingstoke, Hampshire, United Kingdom). Materials for the study, such as demineralized and deionized water and gum arabic (*Acacia senegal* (L.) Willd.) adapted to specific foods, were obtained commercially (Panadia Laboratories).

### The development of spontaneous fermentation in Sumbawa mare’s milk

The fermented mare’s milk had been spontaneously fermenting for approximately 2–3 days during the journey of shipping the samples using land vehicles from Sumbawa, West Nusa Tenggara, to Malang, East Java. During the shipping journey from Sumbawa, mare’s milk, a natural process occurred that resulted from the active microorganisms in the milk due to high temperature, environment, and nutritional factors that fulfill the prerequisite conditions for fermentation. These microorganisms, especially lactic acid bacteria (LAB), will continue to metabolize during the transportation process, converting lactose (milk sugar) into lactic acid. This process will change the physical and chemical properties of milk, such as flavor, aroma, and texture.

According to research by Ganzorig et al. [3], spontaneous fermentation is a very natural and unique process where microorganisms such as LAB that are naturally associated with Sumbawa mare’s milk will always actively convert lactose and other nutrients that make it an energy source into organic compounds without the need for additional enzymes or controlled environmental conditions, so that the taste, aroma, and texture of mare’s milk are different from other milk. The analytical results of spontaneously fermented fresh Sumbawa mare’s milk that serves as a reference for food safety are presented in Table 1.

**Table 1.** Analytical results of spontaneously Sumbawa fermented mare‘s milk compared with literature studies that serve as food safety references.

Test	Reference values	Results
Antibacterial activity test		
*Lactobacillus casei*	ND	4.6 mm
*Bacillus subtilis*	ND	4.75 mm
*Escherichia coli*	Min 8–9 mm [4,5]	9.93 mm
*Pseudomonas aeroginosa*	Min 6–7 mm [3,4]	8.63 mm
Antioxidant activity (IC_50_)	71.5 ppm [12]	62.37 ppm
Total *Escherichia coli* contamination	<3 CFU/ml	0.37 CFU/ml

ND, not detected.

### Research design and preparation of dry fermented mare’s milk Sumbawa

This study was analyzed using a combination of experimental quantitative and qualitative methodologies. The qualitative approach using a completely randomized design was designed as follows. By homogenizing all ingredients and combining 150 ml of mare’s milk with gum arabic at percentages of 20%, 24%, 28%, and 32% + 1% food-grade Tween. The vacuum foam drying oven method was used to dry the fermented mare’s milk. As for the quantitative method, it was reviewed from the content of *E. coli* bacteria and the microstructure of Sumbawa mare’s milk powder.

The research design on making Sumbawa mare’s milk powder on knowing previous research on the composition of ingredients that have been modified. According to Purbasari [11], the results of previous research were developed in the pre-research process with preliminary research by trial and error.

The results of introducing the material composition were obtained as much as 50 ml of Sumbawa mare’s milk + 1% Tween + 5%–11% gum arabic using a vacuum temperature of 70°C for 200 min., with the result that the samples obtained were sticky and could not be processed by grinding. The next stage increased the percentage of volume and gum arabic as much as 350 ml of Sumbawa mare’s milk + 1% Tween + 11% - 20% gum arabic with an increase in time of 420 min. The results obtained were wet samples.

With the results obtained, the material composition was 150 ml of Sumbawa mare’s milk + 1% Tween + 20%–32% (T1–T4) gum arabic with a vacuum oven temperature of 70°C for 420 min. The sample results are in the form of dry flakes and can be processed by grinding. The results of grinding flakes obtained in the form of powder are in accordance with commercial milk powder. Samples were pooled by increasing the proportion of gum arabic as treatments in terms of % w/v.

This study did not use a control because, in the production process by trial and error, it was found that Sumbawa mare’s milk powder without using microencapsulants was damaged due to vacuum heating, so in the production process, a percentage was obtained, which was used as a benchmark with T1:20% – T4:32% with a value of heating vacuum foam drying oven 70°C for 420 min. However, the results of the analysis have been compared by analyzing the fresh product first to determine the standards set to determine the comparison between the reference value and the Sumbawa mare’s milk test, thus providing an assessment of whether the Sumbawa mare’s milk is still suitable for consumption or not [2]. In alternate to the control treatment presented by knowing from the reference factor analysis of spontaneously fermented Sumbawa mare’s milk (Table 1).

### Antibacterial activity analysis (Kirby–Bauer method)

The antibacterial analysis is carried out using the disc diffusion method, which has been evenly dripped with the samples from the previous analysis, followed by testing the antibacterial activity on Gram-positive and Gram-negative bacteria (*L. casei* ATCC 393, *B. subtilis* ATCC 6633, *E. coli* ATCC 25922, and *P. aeruginosa* ATCC 15442). Furthermore, for antibacterial analysis, use nutrient agar media that has been sterilized in an autoclave at 110°C and chilled to the typical standard body temperature of 37°C. The bacteria culture was analyzed by diluting it with BPW to a concentration of 103, then slowly putting it into a Petri plate and waiting for it to form into a gel. Insert a non-antibiotic blank disk (diffusion disk) into a petri dish (up to 4 pieces per replication) and thoroughly submerge in 1000 μl of dry fermented mare’s milk Sumbawa samples on varied treatments. Incubate at 37°C for 24–48 h. After the incubation procedure is completed, a caliper is used to observe and measure the area of the produced clear zone.

### Antioxidant activity analysis (2,2-diphenyl-1-picrylhydrazyl IC_50_)

Antioxidant percentage analysis is often performed by measuring the percentage of inhibition using the 2,2-diphenyl-1-picrylhydrazyl (DPPH) reagent, with modifications. Before doing the antioxidant measurement, the dried sample was diluted and placed in a cuvette container with as many treatments and replicates as required. A spectrophotometer was used to measure the absorbance at the determined wavelength of 517 nm. IC_50_ test with DPPH solution test sample preparation, which was strengthened using a linear equation using the formula *y* = *ax* + *b* [12]. The IC_50_ absorbance values produced by a sample were classified and presented in Table 2.

**Table 2.** The IC_50_ absorbance values.

No	IC_50_	Description
1	<50	Very strong
2	50–100	Strong
3	100–150	Weak
4	150–200	Very weak

### Microbiological analysis (colony-forming unit)

The total *E. coli* test findings for the sample were determined by counting the number of *E. coli* colonies that grew following incubation on a 3M *E. coli/coliform* petrifilm culture medium. To calculate, take a sample of up to 1,000 μl using a micropipette and dilute it with 9 ml of BPW media until it reaches a dilution of 104.

Furthermore, the pouring operation is done slowly on it. The Petrifilm cover was closed and leveled with a spreader. The samples were then kept in an incubator at 37°C for 24 h. After 24 h, the number of bacterial colonies was counted using a colony counter, and samples were marked with a red dot. *E. coli* can live in dried foods that include powdered milk, posing a food safety risk to customers. This is due to the low moisture level in these items, which does not impede the growth of these infections [13].

### Microstructural analysis of dry fermented mare’s milk from Sumbawa using FESEM/EDX.

The morphology was examined using a scanning electron microscope (FESEM, NextSeq 550). The microstructure of dry fermented mare’s milk was investigated, and the sample grains were qualitatively identified and measured in a 60–80 mesh range. The samples’ area maps were analyzed with Image JED-2300 reader software for quantitative analysis utilizing EDX as a function as a reader of the essential elements contained in each particle of Sumbawa mare’s milk powder. The dried fermented mare’s milk Sumbawa samples were quantified by doing phase quantification measurements.

### Protein content analysis of dry fermented Sumbawa mare’s milk using LCMS/MS

The analysis using LCMS/MS can also be utilized as an alternative way to identify and determine the molecular structure of proteins by providing an overview of steroid configurations, aromas, and fragrances displayed graphically. Process samples accurately with an analytical balance of 1–5 gm. Transfer the scaled sample to a special container for LCMS/MS equipment by adding 10–20 ml of 70% EtOH solvent. Homogenized using a vortex for 5–10 min. Centrifuged the sample from the homogenization process at high speed for 10–15 min. Carefully take the supernatant (liquid part) and transfer it to another tube for the reading process by injection into the LCMS/MS device.

### Analysis used by the research

The data collected will be analyzed using analysis of variance and continued with Duncan’s multiple range test, which indicates that there are significant differences between treatments at 1% and 5% significance levels. Analysis of antimicrobial data obtained from dry Sumbawa fermented mare’s milk powder with gum arabic microencapsulation shows the average value ± SD If there is a very real or real difference, the 1% and 5% significance levels will be applied. The last step is to conduct quantitative analysis in descriptive development, namely analysis of *E. coli* content, microstructure with FESEM/EDX, and protein content with LCMS/MS in Sumbawa mare’s milk powder reinforced by literature studies.

## Results and Discussion

### Antibacterial analysis

**Figure 1.**
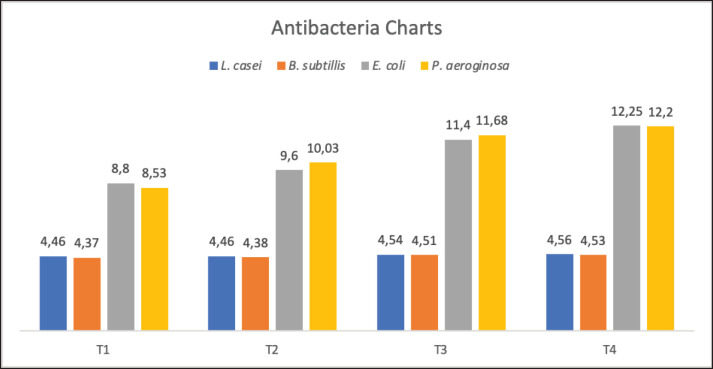
Chart of antibacterial analysis results for Lactobacillus casei (blue), *Bacilus subtillis* (orange), *Escherichia coli* (grey), and *Pseudomonas aeroginosa* (yellow).

Figure 1 shows that the graph of statistical analysis results for *L. casei, B. subtilis, E. coli,* and *P. aeruginosa* from the results of using mycorrhizal microencapsulant gum arabic Sumbawa fermented mare’s milk powder. The results of the analysis obtained and listed in Figure 1 state that the presentation that occurs in the average results obtained by *L. casei* (4.46–4.56 mm), *B. subtilis* (4.37–4.53 mm), *E. coli* (8.8–12.25 mm), and *P. aeruginosa* (8.53–12.2 mm). Some strains of LAB, like *L. casei* and *B. subtilis,* are used as probiotics in foods and supplements. The possibility of the results of antibacterial analysis carried out there are several strains of *Bacillus* sp. that have non-toxic content obtained and used as a source of artificial and natural probiotics in additives in the food industry, including the dairy industry, especially found in Sumbawa mare’s milk.

The purpose of analyzing Gram-positive bacteria that have functional value as probiotics is to support that the probiotic species consisting of several LAB specimens have functionality as antibacterials, whereby testing the clear zone diameter value in the test. It can be indicated that LAB specimens have bacteriocins by degrading the concentration of cell walls in rigid pathogenic bacteria. Where fermentation conditions such as temperature, pH, and nutrient availability will affect the production of bacteriocin [6,12].

Based on the comparison of the analysis results of Gram-negative bacteria testing, such as *E. coli* and *P. aeruginosa*, Sumbawa fermented mare’s milk powder with gum arabic microencapsulant has the capability as an antibacterial agent based on the analysis results from T4 with a range of clear zone diameter values in the inhibition of high pathogenic bacteria (Fig. 1). These results confirm that gum arabic does not necessarily protect important substances in Sumbawa fermented mare’s milk powder but has an important role in protecting it from damage during the heat drying process, as can be ascertained from the test results of the inhibition zone of the pathogenic bacteria. Gum arabic as a microencapsulant has important roles, such as cell wall formation: it forms a protective wall around bacteriocins and LAB, protecting them from physical and chemical damage. Release controller: It can control the release rate of bacteriocins and LAB. Increased stability: It improves the stability of bacteriocins and LAB against temperature, pH, and digestive enzymes of Sumbawa fermented mare’s milk powder, and Increased solubility: It can increase the solubility of bacteriocins and LAB in powdered dairy products, thus facilitating dispersion and even distribution [7,13].

Family Lactobacilli and Bacillus are known as one of the Gram-positive bacteria that perform fermentative processes in fresh milk, especially Sumbawa mare’s milk, by performing a decay process by degrading lactose, or milk sugar, into lactic acid, acetic acid, and several other organic acids that can be producers of bacteriocins, namely antibacterials. It can be identified that the strains of the bacterial subspecies are positive bacteria, which are an example of probiotics in the field of health science sourced from dairy products [14].

According to the results of the analysis of Chugh et al. [15], the antibacterial content produced by LAB against pathogenic bacteria *E. coli* has a high inhibition of 13.05 mm, while according to Alipour et al. [16], the inhibition of fermented milk against pathogenic bacteria was 11.34 mm and included a high class. It is that *E. coli* bacteria can be inhibited due to the antibacterial activity of fermented milk, such as Sumbawa mare’s milk and its processed products.

These pathogenic bacteria (*E. coli* and *P. aeruginosa*) are some of the pathogenic bacteria that can develop during the storage process [17], but in the results of the analysis carried out, there was an increase in the inhibition zone, especially in T4 (*p* < 0.01), which was initiated by the increased use of gum arabic as a microencapsulant that protects the activity of LAB. Antibacterial components are produced during lactose fermentation by LAB, such as organic acids (lactic acid and acetic acid), carbon dioxide, hydrogen peroxide, ethanol, diacetyl, and peptides (bacteriocins) [15].

### Antioxidant activity analysis (DPPH IC_50_)

The test results that have been carried out according to the T1–T4 (*p* < 0.01) treatment of Sumbawa horse milk powder with the use of gum arabic as a microencapsulant show that the higher the antioxidant activity based on the IC_50_ value. The antioxidant content of each treatment T1: 62.20 ppm - T4: 51.78 ppm, from the results of the tests that have been carried out, it can be seen that the use of gum arabic microencapsulant in the manufacture of Sumbawa fermented mare’s milk powder will provide an alternative additional ingredient as a probiotic food reserve that will increase the activation process of bioactive substances from fermentation that can produce antioxidant activity. According to research results from Sik et al. [12], antioxidant activity results from the breakdown of milk proteins such as lactoferrin, lactoglobulin, k-casein, and immunoglobulin. These proteins are derived from bioactive peptides consisting of hydrophobic amino acids proline, histidine, and tyrosine. In the sequence, there is a process of amino acid hydrolysis due to proteolytic enzymes used in the fermentation process by LAB that are working. The test results of the antioxidant of Sumbawa fermented mare’s milk powder are presented in Table 3 and compared with Table 2. The antioxidant content will be stronger if the IC_50_ value in ppm (< 50 ppm); the lower the IC_50_ value, the stronger the antioxidant activity. Otherwise, if the IC_50_ value exceeds >150 ppm, the activity is weaker.

**Table 3.** The test results of antioxidant of Sumbawa fermented mare‘s milk powder.

Treatment	Average IC_50_ (ppm)
T1	62.20 ± 1.81^c^
T2	58.55 ± 0.82^b^
T3	56.52 ± 0.84^b^
T4	51.78 ± 1.97^a^

a,b,c The superscripts presented indicate significant differences (*p* < 0.01) in the mean IC_50_ antioxidant activity values of Sumbawa horse milk powder with gum arabic microencapsulant.

**Table 4.** Microbiological analysis *Escherichia coli* Sumbawa fermented mare’s milk powder.

No.	Treatment	Count of *Escherichia coli* bacteria (CFU/gm)	Standardized numbers (CFU/gm)
1.	T1	0.33	<3
2.	T2	0.08	<3
3.	T3	0	<3
4.	T4	0	<3

The results of the analysis of the number of pathogenic bacteria *Escherichia coli* of Sumbawa horse milk powder obtained that T1–T4 meet the requirements of food quality.

It can be seen that the antioxidant activity with an IC_50_ inhibitory value produced from fermented mare’s milk probiotic is 62.01 ppm, while for spontaneously fermented mare’s milk, koumiss, and shubat have strong antioxidant activity with a range between 50 and 80 ppm, but if added with microencapsulants, it can be ascertained that the stronger the antioxidant content [18,19]. The addition of stabilizers and microencapsulants can improve the suspension by helping to homogenize and disperse milk particles due to the drying process with high thermal conditions, thereby increasing the durability, which can protect milk powder products from oxidation and unwanted chemical reaction damage. Gum arabic, which is a type of polysaccharide that contains polyphenols, can be used as an additive and increase antioxidant compounds in body cells from free radical damage and avoid body inflammation [20]. Based on the results of the research that has been done, it can be seen that the addition of gum arabic T4 (*p* < 0.01) provides a high antioxidant content, and the use of gum arabic, a polysaccharide containing polyphenols, can be used as an additional ingredient to increase antioxidant compounds in body cells from free radical damage and prevent inflammation in the body, especially in Sumbawa fermented mare’s milk powder.

### Microbiological analysis of E. coli

The quality of milk can be determined by the types of microbes present in the milk, which can indirectly affect the palatability and shelf life of milk for consumption. Bacterial contamination in high numbers is inseparable from environmental conditions that support the process of increasing pathogenic bacteria like *E. coli* bacteria exposed to food and beverage samples, using a calculation method with units of CFU/gm, which has been set by international standards with reference to the U.S.A Food and Drug Administration must have a value below 3 CFU/gm [16]. Although positive, the underlying thing is exposure to environmental factors during the manufacturing process and contamination of samples in the equipment environment. The results of the microbiological analysis of *E. coli in* Sumbawa fermented mare’s milk powder obtained in this study can be reviewed in Table 4.

The results of the research that has been carried out have decreased the amount of *E*. *coli* content in each treatment tested, where the higher the use of gum arabic T1–T4 with a value of 0.33–0 CFU/gm, while the results of the analysis state that the specific minimum standard of *E. coli* content in traditional fermented milk preparations is a maximum of <3 CFU/gm, and according to the Codex Alimentarius International standard, the minimum standard of milk powder, fermented milk, and processed milk products is <3CFU/gm. The decrease in the number of *E. coli* bacteria was due to the effect of competition for nutrients and space with other LAB present in Sumbawa fermented mare’s milk powder, which was protected by gum arabic so that LAB produced unfavorable environmental conditions for *E. coli* [21]. Comparable to the research of Fazilah et al. [22], it was stated that gum arabic is one of the backup energy sources needed by probiotics; the high content of polysaccharides and glycoproteins can be used as a cross-contamination protection material from bacteria. Gum arabic is an excellent emulsifier. It can help to stabilize the emulsion and prevent it from breaking down, reducing the risk of contamination. It has been shown to have antibacterial properties, which can help to inhibit the growth of bacteria.

### Microstructure and chemical element content analysis of Sumbawa fermented mare’s milk powder using FESEM/EDX

**Figure 2.**
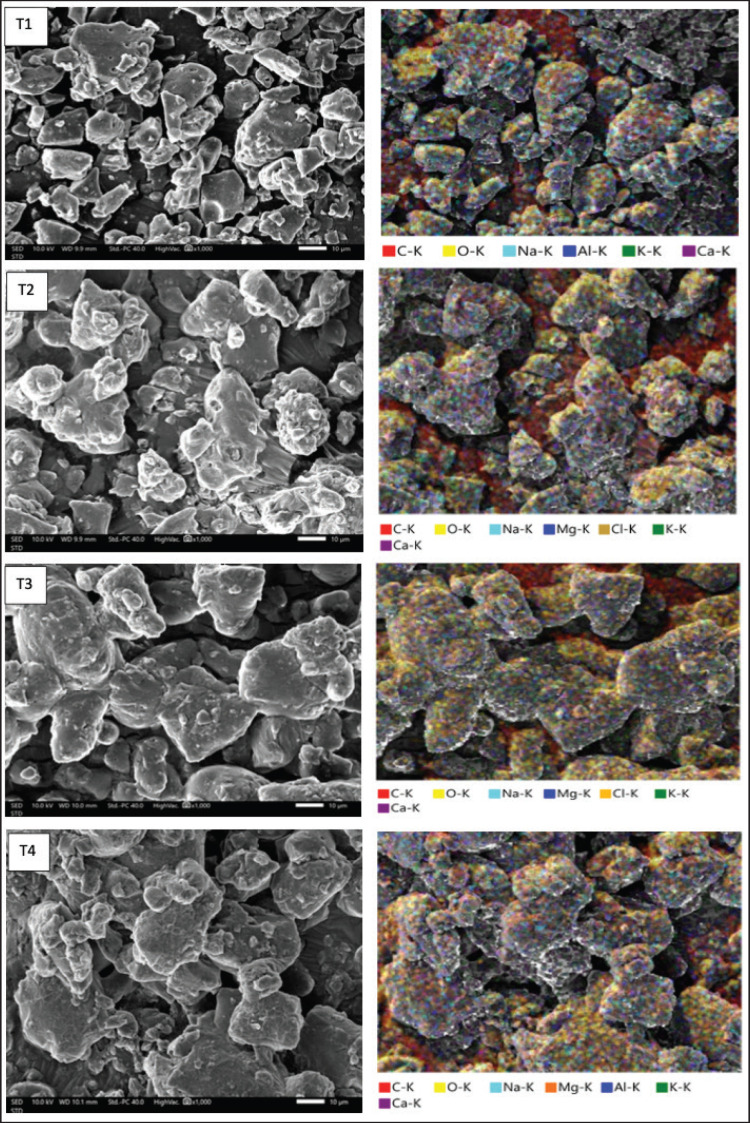
Microstructure and chemical elements content results of T1–T4 Sumbawa fermented mare’s milk powder with gum Arabic (*Acacia senegal*) microencapsulant by FESEM/EDX analysis.

The microstructure test showed some changes in the matrix structure with increasing concentration of Sumbawa horse milk by displaying a more continuous surface size that is associated with the bioactive content of milk with the capacity to use gum arabic emulsifiers used as the main ingredient of microencapsulants. According to the findings reported by Magnini et al. [23], the size of microstructural observations of a sample ranging from 10 μm can be a reference and a key role in reading the condition and content of a sample being analyzed. Particle analysis of Sumbawa mare’s milk powder T1–T4 showed a distribution of changes in particle size from small to large according to the addition of gum arabic as a microencapsulant of Sumbawa horse milk powder. This was attributed to the stronger protein binding done by gum arabic during the drying process. The structure of Sumbawa fermented mare’s milk powder (Fig. 2) observed by FESEM is typical of powders dried by the vacuum foam drying method.

The compactness of the milk powder particle structure is critical as it can affect various functional properties and the quality of the product to be produced. A compact particle structure and evenly distributed porosity have a significant influence on the rehydration, solubility, stability, and texture of the product [24]. Conversely, if the porosity between pores is uneven or too much. By microencapsulating gum arabic, we can determine the capability of the particles in increasing the solubility of the active ingredients in water. In addition, good particle cohesiveness can help in faster and more complete disintegration when milk powder is dissolved. The results of the analysis showed that the increasing percentage of gum arabic T1–T4 as microencapsulant had a controlling effect on pore size and porosity, as shown in the blue-colored circle. Increasing gum arabic in the microencapsulation formulation can produce particles with more uniform and controlled pores. The gel-forming structure of gum arabic allows for a denser and more organized matrix to form, which in turn reduces total porosity but promotes a more even pore size distribution [25].

The chemical elements contained in Sumbawa horse milk powder have various functions for the stability of nutrients in the sample. This can be analyzed using EDX, which functions as a reader of the important elements contained in each particle of Sumbawa horse milk powder. EDX provides a way of identifying the chemical elements contained in Sumbawa horse milk powder, such as C, O, Na, Al, Cl, K, and Ca. All of this information is essential for understanding the nutritional profile and quality of the milk. The chemical element readings of Sumbawa horse milk powder provided information that calcium (Ca) may play a role in enhancing antibacterial activity through milk processing and preservation. Magnesium (Mg) plays an important role in several biochemical processes, including the synthesis and activity of antibacterial-related enzymes. The sodium compound Na can act as an interaction material with organic acids such as lactic acid and acetic acid produced during the fermentation process, thus helping to maintain pH stability [25,26]. The EDX reading results of the chemical element content of Sumbawa fermented mare’s milk powder are shown in Table 5.

**Table 5.** The EDX reading results of the chemical element content of Sumbawa fermented mare‘s milk powder.

No.	Chemical element	Chemical content concentration			
		T1	T2	T3	T4
1.	Al	0.037	0	0	0.147
2.	C	64.857	65.173	60.647	62.713
3.	Ca	0.477	0.353	0.503	0.583
4.	Cl	0	0.113	0.160	0
5.	K	0.237	0.200	0.270	0.307
6.	Mg	0	0.240	0.163	0.273
7.	Na	0.333	0.243	0.343	0.273
8.	O	34.057	33.680	37.923	35.730

Sodium compounds can help improve the efficiency of nutrient absorption by the body, especially calcium and protein. The elements oxygen (O) and carbon (C) in Sumbawa mare’s milk powder provide information on the composition, quality, and nutritional value of the product. Comparable to the analysis results of Cais-Sokolińska et al. [26], some elements of Mg, Al, and Ca have functions as cofactor agents in the antibacterial activation of LAB against pathogenic bacteria involving various interrelated processes, including enzyme activation, cell structure stability, inhibition of pathogenic bacterial growth, and food preservation. The increasing addition of gum arabic (T1–T4) to Sumbawa milk in food products can contribute significantly to the chemical composition, including the increase of certain atomic elements in the product samples. Gum arabic is a complex polysaccharide extracted from the sap of the acacia tree and consists of various sugars, including arabinose, galactose, rhamnose, and glucuronate, as well as minerals that exert an adding influence on the C and O chemical atom chains.

### Pharmaceutical activities and protein components of Sumbawa fermented mare’s milk powder with LCMS/MS analysis

The protein component and pharmaceutical properties of traditional health-beneficial beverages derived from Sumbawa mare’s milk powder are presented comprehensively, providing more precise specific details, which will be outlined in Table 6, by presenting several studies and a comprehensive overview of preclinical investigations conducted to test the health-beneficial properties of Sumbawa mare’s milk before and after processing.

**Table 6.** The summary of proteinpharmaceutical effectiveness content of traditional beverages derived from Sumbawa fermented mare’s milk powder.

Pharmaceutical activities	Beneficial compounds (proteins) from LCMS/MS analysis	Result	Reference from the result of benefical compounds
Anti-hypertensive	ACE peptides inhibitors, k-Casein	Reducing the hormone angiotensin II by the performance of ACE inhibitors will lower blood pressure and ease the work of the heart. this drug compound can also help to overcome damage from kidney activity	[18,29]
Antibacteria	Lysozyme, lactoferrin and lactalbumin peptides	Increase in beneficial bacterial variants of the genus *Lactobacillus* (Lb.) acts an important role in influencing the bacteriosins functionality of pathogens a lot of attention is focused on the safety of these strains, due to probiotic properties and further enhancing the fermentation process of lactose in order to increase antimicrobial peptides (citric, lactic, and malic acids belong to the first group, and acetic acid should be considered an ethanol biotransformative).	[3,27,28,30]
Antioxidant	Lactoferrin peptides	One of the peptides that has the ability to counteract the so-called explosion of oxygen reception in neutrophils, which will result in a large production of free radicals by damaging cells in the body's tissues.	[12,13,31]
Anti-alergenicity protein	Lactoferrin, lactoperoksidase, and lactoglobulin peptides	The high lactose in horse milk will be fermented by beneficial bacteria such as lactobacillus, and converted into lactic acid, ethanol and carbon dioxide, with the function of fermenting milk that contains many nutrients, making it accessible to people who are lactose intolerant.	[13,16,21]
AntiInflamatory	α-lactalbumin, β-lactoglobulin, αs1-casein, and κ-Casein peptides	The total nutrient content present in fermented milk, especially horse milk, can increase total IgA and IgG, while reducing total IgE levels analyzed in the blood serum of experimental animals, whey content changes the Th1/Th2 balance towards a Th1 response which will have an immunomodulatory effect on the body thereby reducing inflammation.	[21,25,30,31]

## Conclusion

The final results in this study presented from the treatment used with the addition of gum arabic T4 (32%) as a functional healthy food microencapsulation material of Sumbawa fermented mare’s milk powder have the potential to protect the nutritional and probiotic content so that it can activate its antibacterial content; besides that, adding gum arabic can actively control the antioxidant content. The results of this study will improve the quality of milk powder in terms of physical conditions in microstructure, where the use of gum arabic microencapsulant (T4) provides significant changes in terms of increasing the percentage of the use of gum arabic in microencapsulating milk powder can provide better stability, more evenly distributed pores, and higher particle compactness. The research conducted will be strengthened by the results of protein content analysis, which pharmacologically reinforces that the Sumbawa fermented mare’s milk powder has the potential to be a healthy processed product. This study highlights the potential of the researched product as a traditional, modified healthy food product option and offers the practicality of the product to be consumed by the general public.

## List of abbreviations

%w/v, weight per volume

μl, microliter

BPW, buffered peptone water

CFU/gm, colony forming unit per gram

DPPH, 2,2-diphenyl-1-picrylhydrazyl

FESEM, field emission scanning electron microscopy with energy dispersive X-ray spectroscopy

gm, gram

LAB, lactic acid bacteria

LCMS/MS, liquid chromatography-mass spectrometry/mass spectrometry

mm, millimeter

T, treatment.
